# Prevalence and associated factors of retinal vein occlusion in the Korean National Health and Nutritional Examination Survey, 2008–2012: A cross-sectional observational study

**DOI:** 10.1097/MD.0000000000005185

**Published:** 2016-11-04

**Authors:** Yong Un Shin, Heeyoon Cho, Jong Min Kim, Kunho Bae, Min ho Kang, Jae Pil Shin, Eunwoo Nam, Se Woong Kang

**Affiliations:** a Department of Ophthalmology, Hanyang University College of Medicine; b Department of Ophthalmology, Samsung Medical Center, Sungkyunkwan University School of Medicine, Seoul; c Department of Ophthalmology, Kyungpook National University School of Medicine, Daegu; d Biostatistical Consulting and Research Lab, Hanyang University, Seoul, Korea.

**Keywords:** epidemiology, hypertension, Korean, retinal vein occlusion

## Abstract

Retinal vein occlusion (RVO) is the second most common retinal vascular diseases and there are only a few Asian population-based studies with small samples. Hypertension is one of a modifiable risk factor of RVO, but no recent studies have shown the relationship between RVO and hypertension control status. We aimed to investigate the prevalence of RVO and its associated factors in an adult Korean population.

A nationwide population-based, cross-sectional study. We enrolled 37,982 participants from the Korea National Health and Nutrition Examination Survey who were 19 years or older and who had undergone ophthalmologic exams from 2008 through 2012. All participants underwent a comprehensive ophthalmic examination, standardized ophthalmic and health interviews, and laboratory investigations. Digital fundus photographs were interpreted by retinal specialists who investigated for the presence of RVO. The prevalence of RVO was then estimated. RVO-associated factors were determined using step-wise logistic regression analyses. We also performed a subgroup analysis to evaluate the association between hypertension and RVO according to hypertension control status and antihypertensive medication use.

Of those enrolled participants, 25,765 participants met our study criteria and were included in the analyses. The overall RVO prevalence (n = 205) was 0.6 ± 0.1% (0.6 ± 0.1% for branch RVO and <0.1% for central RVO), and no sex differences were observed. In multivariate logistic regression analyses after adjusting for all potential risk factors, we found the following factors to be significantly associated with RVO: old age (odds ratio (OR) = 1.72, 95% CI: 1.27–2.34), hypertension (OR = 2.56, 95% CI: 1.31–5.08), history of stroke (OR = 2.08, 95% CI: 1.01–4.45), and hypercholesterolemia (OR = 1.84, 95% CI: 1.01–3.35). In a subset of participants with hypertension, participants with uncontrolled hypertension (OR = 3.46, 95% CI: 1.72–6.94) and unmedicated hypertension (OR = 4.12, 95% CI: 2.01–8.46) were more significantly associated with RVO than participants without hypertension.

RVO prevalence in Korea was moderate relative to that in the rest of the world, and RVO-associated factors were similar to those identified in other population-based studies. Well-controlled hypertension and antihypertensive medication showed inverse association with RVO.

## 1 Introduction

Retinal vein occlusion (RVO) is the second most common retinal vascular diseases following diabetic retinopathy. RVO causes macular edema or vitreous hemorrhage and can lead to visual disturbance.^[1–3]^ RVO pathogenesis is generally due to compression of the venous lumen by arterial hemodynamic alterations and/or inflammation.^[4]^ RVO prevalence ranges from 0.3% to 1.6% according to several reports, including cohort studies, and some have suggested that RVO is associated with either systemic disorders (such as hypertension or diabetes) or ophthalmic disorders (such as ocular hypertension or glaucoma).^[1,5–13]^ These data were mostly obtained from epidemiologic studies in Western populations, while data from Asian samples remain insufficient for robust analyses; most Asia-based studies included fewer than 100 subjects.^[7,8,10,12,13]^

Hypertension is one of the major cardiovascular risk factors and is usually managed with antihypertensive drugs and/or lifestyle modification.^[14]^ Most epidemiological studies agree that hypertension is an important RVO risk factor. However, little is known about whether hypertension control and antihypertensive medication reduce RVO risk.^[1]^

The purpose of this study was to use a nationwide health survey to investigate the prevalence of RVO according to age and sex and to identify possible risk factors of RVO, especially when hypertension is being controlled with or without antihypertensive medication.

## 2 Methods

### 2.1 Study design and population

Data from the Korea National Health and Nutrition Examination Survey (KNHANES) were reviewed for this study. The KNHANES is an ongoing cross-sectional, nationwide, population-based survey of the health and nutritional status of noninstitutionalized South Korean people, beginning in 1998. It is based on a complex, stratified, multistage, clustered probability design in order to obtain a representative sample of the Korean population. Details of the KNHANES sample recruitment strategy have been described elsewhere.^[15–17]^ The KNHANES consists of 3 parts: the Health Interview Survey, the Health Examination Survey, and the Nutritional Survey. The ophthalmologic interviews and exams were only conducted from July 2008 to December 2012. Of the 45,810 participants enrolled over the 5-year period in which ophthalmologic data were collected, 37,982 participants participated in the ophthalmologic survey. Fundus photography was performed only in adults aged 19 years or older; therefore, we included participants ≥19 years old who underwent ophthalmologic interviews and exams as well as general health interviews and exams. Participants were excluded from our analyses if they did not have gradable fundus photographs due to poor image quality or were missing examination or interview data. However, participants who had unilateral RVO but their unreadable fundus photograph corresponded to the unaffected eye were included in the study. All participants provided written informed consent, and this study design was reviewed and approved by the Institutional Review Board (IRB) of the Korean Centers for Diseases Control and Prevention and by the IRB of Hanyang University Guri Hospital.

### 2.2 Data collection

We selected possible risk factors from the KNHANES open-access data based on previous RVO epidemiologic studies. The health interview survey consisted of standardized questionnaires including demographic and socioeconomic information and current or previous medical conditions. Monthly household income (quartiles of household income) and education level (elementary school or less, middle or high school, college or more) were collected as socioeconomic factors. Patients were categorized into 1 of 2 smoking statuses: either current smoker (a lifetime history of smoking more than 5 packs of cigarettes or smoking at the time of the interview) or nonsmoker (all categories of smoking other than current smoker). Patients were categorized into 1 of 2 alcohol-drinking statuses: regular alcohol drinker (currently drinking alcohol more than once per month) or nondrinker (all categories of alcohol drinking other than regular alcohol drinker). Individual medical histories were obtained by self-reported questionnaires, including data on history of angina, myocardial infarction, and stroke. However, other individual medical histories, such as hypertension or diabetes, were not used in this study.

The health examination survey included anthropometric data, blood pressure, and biochemical data. Waist circumference, height, and weight of participants were measured by specifically trained examiners. Body mass index (BMI) was calculated by dividing weight in kilograms by height in meters squared (kg/m^2^). Blood pressure was measured 3 times on the right arm after at least 5 minutes of rest in a seated position (Baumanometer; W.A. Baum Co., Copiague, NY). We calculated the final blood pressure value by averaging the second and third blood pressure measurements. For the routine blood test, blood samples were collected after at least an 8-hour fasting period and were analyzed within 24 hours after transport to a certified laboratory. From the blood sera data, fasting glucose, glycated hemoglobin (HbA1c), total cholesterol, triglycerides, high-density lipoprotein, low-density lipoprotein, white blood cell count, hematocrit, ferritin, vitamin D, blood urea nitrogen, and creatinine were all analyzed for this study.

Ophthalmology-focused interviews were performed using self-reported questionnaires, including past or current medical or surgical conditions relevant to ophthalmology, including history of cataract surgery. Exams were performed by an ophthalmologist who had been periodically trained and certified by the Korean Ophthalmological Society (KOS) National Epidemiologic Survey Committee. Intraocular pressure (Goldmann applanation tonometry) and refractive errors (automatic refractometry, KR-8800; Topcon, Tokyo, Japan) were measured. Slit-lamp biomicroscopy was performed to identify any anterior segment abnormalities and to measure the chamber depth. Nonmydriatic 45° digital fundus photography (TRC-NW6S; Topcon) was performed on patients who participated in the ophthalmologic exams and were 19 years or older. If participants had a history of diabetes, random glucose level higher than 200 mg/dL, or suspicious diabetic retinopathy findings on nonmydriatic fundus photography, 7 standard field photographs were obtained after pharmacological pupil dilation. Automated visual field testing using the screening program N-30-1 (Humphrey Matrix frequency-doubling perimeter; Carl Zeiss Meditec, Inc., Dublin, CA) was performed on participants who had elevated intraocular pressure (≥22 mm Hg), a horizontal or vertical cup to disc ratio ≥0.5, violation of the ISNT rule (neuroretinal rim broadest in the inferior (I) area in the normal eye, followed by the superior (S), nasal (N), and temporal (T) areas), an optic disc hemorrhage, or a retinal nerve fiber layer (RNFL) defect. Each fundus image was preevaluated onsite by ophthalmologists at the time of the examination (normal vs abnormal) and all images were sent to a central reading center and were evaluated preliminarily by nine retinal specialists who participated in the Epidemiologic Survey Committee of the KOS. Final grading was determined by 1 retinal specialist (J.P.S.) after resolving interpreting discrepancies. RVO was categorized as branch RVO (BRVO) and central RVO (CRVO) and defined according to a standardized protocol proposed in previous studies.^[1,5,6]^ Recent CRVO was characterized by retinal edema, optic disc hyperemia or edema, scattered superficial and deep retinal hemorrhages, and venous dilation. Old CRVOs were characterized by occluded and sheathed retinal veins or vascular anastomosis at the optic disc. BRVOs involved a localized area of the retina in the sector of the obstructed venules and were characterized by scattered superficial and deep retinal hemorrhages, venous dilation, intraretinal microvascular abnormalities, and occluded and sheathed retinal venules. A patient was determined to have RVO if either of the eyes had BRVO or CRVO. For analyzing eye-specific factors such as glaucoma, refractive errors, history of cataract operation, and ocular perfusion pressure, we obtained ocular information from 1 eye with RVO. Even in cases with bilateral RVOs, ocular data from only 1 eye (right eye) was selected for analysis.

### 2.3 Variable definitions

Several new variables were defined for this study from the KNHANES raw data. Hypertension presence was defined as systolic pressure >140 mm Hg, diastolic pressure >90 mm Hg, or a current prescription for antihypertensive medication. Diabetes presence was defined as fasting glucose >126 mg/dL or a current prescription for antiglycemic medication. Hypercholesterolemia was defined as a total cholesterol concentration >240 mg/dL or a current prescription for anticholesterol medication. Pulse pressure was defined as the difference between the systolic and diastolic pressure measurements. Metabolic syndrome was defined using previously known criteria proposed by the International Diabetes Federation in 2009.^[18]^ We identified cases of chronic kidney disease (CKD) by calculating the eGFR (estimated glomerular filtration rate) using the Modification of Diet in Renal Diseases Study formula: eGFR = 186.3 × (serum creatinine)^−1.154^ × age^−0.203^ × 0.742 (for women).^[19]^ CKD was defined as eGFR value <60 mL/min/1.73 m^2^.^[20]^ Glaucoma was defined using the International Society of Geographical and Epidemiological Ophthalmology classification criteria. Category 1 requires both a visual field defect consistent with glaucoma as well as a vertical cup-to-disc ratio (VCDR) ≥0.7, asymmetry of the VCDR ≥0.2, or presence of an RNFL defect; category 2 (when the visual field test was inconclusive) required VCDR ≥0.9, asymmetry of the VCDR ≥0.3, or presence of an RNFL defect with violation of the ISNT rule; category 3 (when no visual field testing or optic disc examination was available) required a visual acuity <20/400 and an intraocular pressure greater than 21 mm Hg.^[21]^ ocular perfusion pressure was determined as two-thirds of the mean arterial blood pressure (two-thirds of the diastolic plus one-third of the systolic value) minus the intraocular pressure.

### 2.4 Subgroup analysis

To analyze the association between hypertension and RVO in detail, participants with hypertension were divided according to hypertension control status and antihypertensive medication use.^[14]^ Stage 1 hypertension was defined as systolic pressure >140 mm Hg or diastolic pressure >90 mm Hg. Stage 2 hypertension was defined as systolic pressure >160 mm Hg or diastolic pressure >100 mm Hg. Controlled hypertension was defined as systolic pressure ≤140 mm Hg and diastolic pressure ≤90 mm Hg among patients taking antihypertensive medication. Uncontrolled hypertension was defined as systolic pressure >140 mm Hg or diastolic pressure >90 mm Hg among patients taking antihypertensive medication. We analyzed the association between RVO and hypertension control status regardless of antihypertensive medication use. We also compared the association of RVO between participants with and without antihypertensive medication use.

### 2.5 Statistical analysis

Statistical analyses for a complex sampling design were performed using SPSS for Windows software, version 18.0 (SPSS, Inc., Chicago, IL). According to the statistical guideline from the Korea Centers for Disease Control and Prevention, we organized a new dataset integrating the 5-year data and applied adjusted weights. Baseline characteristics of enrolled participants are presented as mean ± standard error (SE) for continuous variables and as percentage (%) ± SE for categorical variables and were compared using the independent *T* test and the Chi-square test, respectively. Based on the difference between baseline characteristics of RVO and non-RVO participants, we selected potential risk factors with a *P*-value <0.1 for logistic regression analyses. A step-wise approach was used to determine which factors had significant associations with RVO. In first step, simple linear regression analyses were performed to identify associations between risk factors and RVO. Factors associated with an increased RVO risk with a *P*-value <0.1 were entered into multivariate logistic regression analyses. In next step, we calculated odds ratios (OR) and 95% confidence intervals (CI) after adjusting for age and all other confounders. Factors that yielded a *P*-value ≤0.05 were considered statistically significant.

## 3 Results

**Figure 1:**
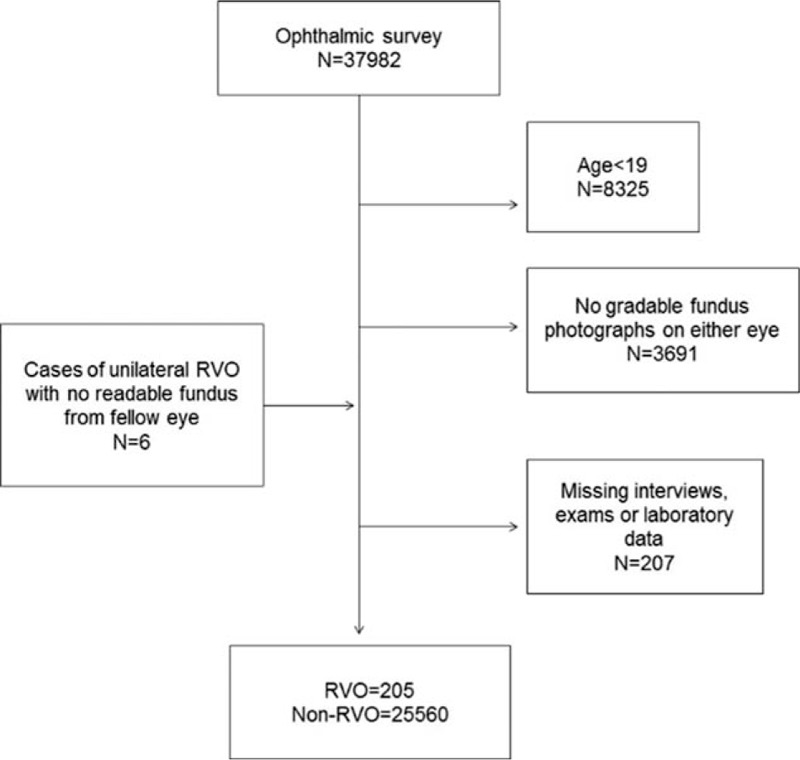
Flow chart for selection of study participants.

Of all the participants who underwent an ophthalmic survey (N = 37,982), 25,765 were eligible for this study (205 participants with RVO and 25,560 participants without RVO). We excluded 12,223 participants because they were younger than 19 years; had a poor-quality, ungradable fundus image on either eye; or were missing survey data. Participants with RVO in 1 eye but whose unreadable fundus was for the fellow eye were included (6 participants) (Fig. 1).

### 3.1 Prevalence of RVO

The overall prevalence of any type of RVO was 0.6 ± 0.1% in the Korean population older than 19 years. There was no significant difference in RVO prevalence between males and females (0.6 ± 0.1% in both sexes). When we limited our analyses to participants older than 40 years, the prevalence increased to 1.0 ± 0.1%. BRVO prevalence was 0.6 ± 0.1% (80 male participants, 117 female participants), whereas CRVO prevalence was much lower at 0.1% (3 male participants and 6 female participants); again there was no prevalence difference for either subtype by sex. RVO was rarely observed in participants younger than 40 years, and no CRVO was found in participants younger than 60 years (Table 1).

**Table 1:**
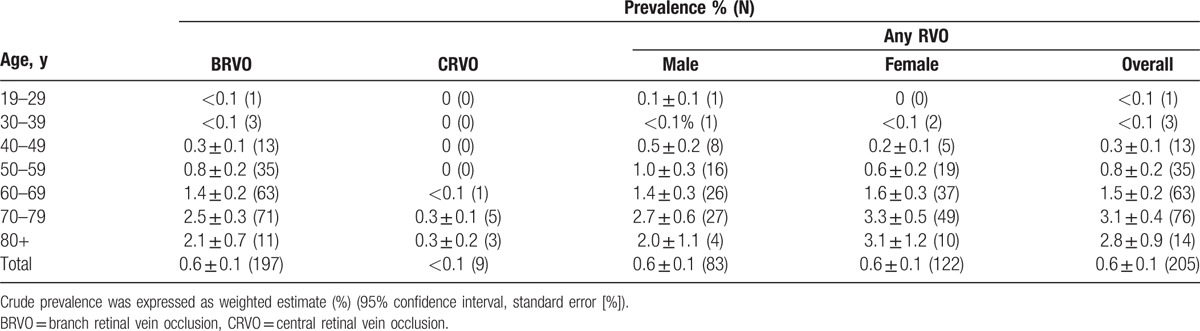
Prevalence of retinal vein occlusion according to age, sex, and retinal vein occlusion subtype.

### 3.2 Risk factors associated with RVO

Regarding sociodemographic factors, participants with RVO were more likely to be older (62.7 ± 1.2 vs 44.3 ± 0.2 years, *P* < 0.001), have a lower household income (41.7 ± 4.3 vs 20.0 ± 0.5% in proportion of lower quartile, *P* < 0.001), and have a lower education level (53.1 ± 4.3 vs 30.4 ± 0.6% in proportion of high school or less, *P* < 0.001) than participants without RVO. General medical conditions, such as the presence of diabetes (14.6 ± 3.5 vs 7.7 ± 0.2%, *P* = 0.011), hypertension (70.2 ± 3.8 vs 25.6 ± 0.4%, *P* < 0.001), CKD (5.8 ± 1.7 vs 1.5 ± 0.1%, *P* = 0.010), and previous stroke (7.5 ± 1.8 vs 1.2 ± 0.1%, *P* < 0.001), were more frequent among participants with RVO than those without. Regarding the biochemical factors, fasting glucose (103.1 ± 1.9 vs 96.4 ± 0.2 mg/dL, *P* = 0.026) and total cholesterol (199.9 ± 4.2 vs 187.0 ± 0.3 mg/dL, *P* = 0.004) were significantly higher in participants with RVO than in those without RVO. Glaucoma (14.6 ± 3.2 vs 5.3 ± 0.2%, *P* < 0.001), higher ocular perfusion pressure (49.0 ± 3.8 vs 37.4 ± 1.1 mm Hg, *P* = 0.005), history of cataract surgery (2.1 ± 0.9 vs 0.8 ± 0.1%, *P* = 0.018), and hyperopic refractive errors (0.9 ± 0.2 vs −0.4 ± 0.0 diopters, *P* < 0.001) were more frequently observed in participants with RVO than in those without RVO. Detailed baseline characteristics are shown in Table 2.

**Table 2:**
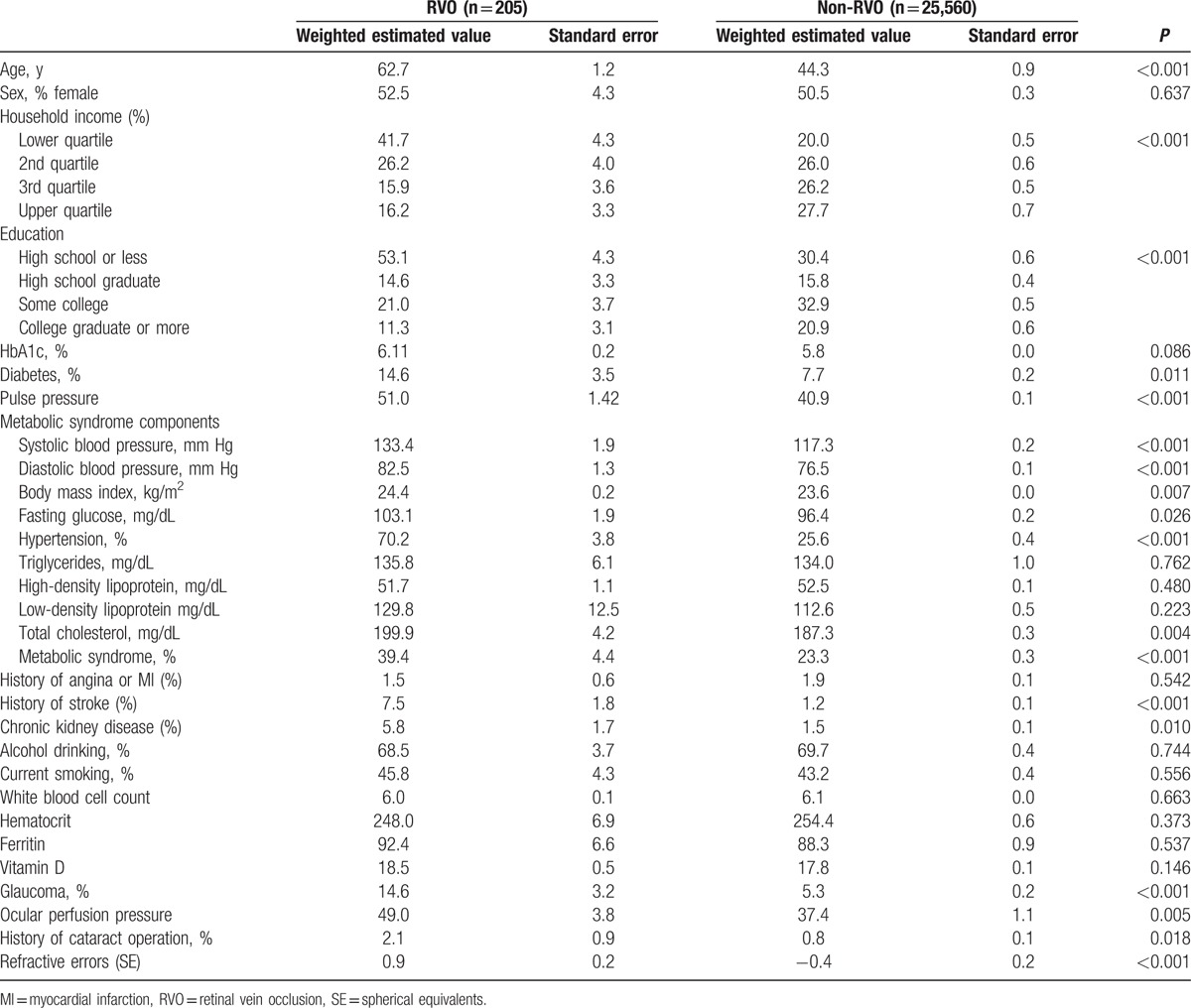
Comparison of characteristics between participants with and without retinal vein occlusion.

Table 3 shows the RVO-associated factors as determined by logistic regression analysis. According to univariate logistic regression analysis, the following factors were significantly associated with RVO (*P* < 0.01): age, household income, education level, HbA1c, diabetes, pulse pressure, BMI, fasting glucose, hypertension, hypercholesterolemia, history of stroke, CKD, glaucoma, history of cataract operation, and refractive errors. Metabolic syndrome was statistically significant as a risk factor in univariate analysis (OR = 2.14, 95% CI: 1.49–3.07) but was not significant in age-adjusted multivariate logistic regression analysis (age-adjusted OR [aOR] = 1.08, 95% CI: 0.74–1.56). We excluded metabolic syndrome from the multivariate logistic regression analysis because the definition of metabolic syndrome included various components that overlapped with other risk factors. According to our step-wise multivariate logistic regression analysis, old age (aOR = 1.72, 95% CI: 1.27–2.34), hypertension (aOR = 2.58, 95% CI: 1.31–5.08), history of stroke (aOR = 2.08 95% CI: 1.01–4.45), and hypercholesterolemia (aOR = 1.84, 95% CI: 1.01–3.35) were associated with RVO after adjusting for all potential confounding factors.

**Table 3:**
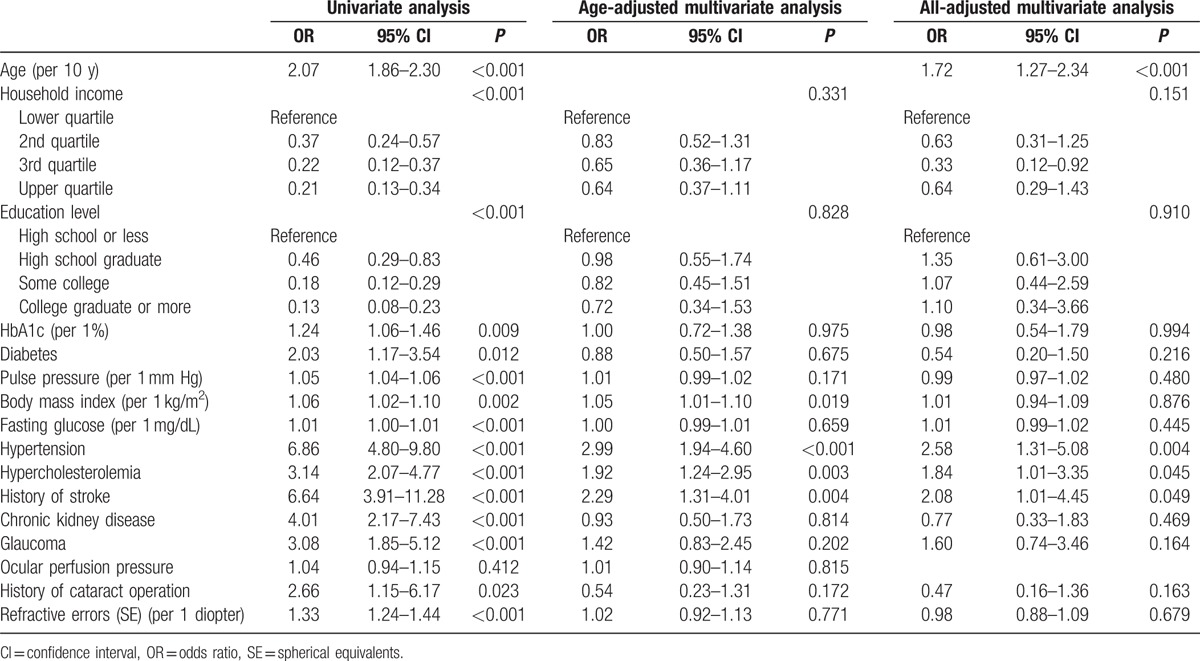
Logistic regression analyses of associations between potential risk factors and retinal vein occlusion.

### 3.3 Association of RVO with hypertension control and antihypertensive medication

Table 4 shows RVO associations according to degree of hypertension, regardless of whether the patient was taking hypertension medication. Multivariate logistic regression analysis with adjustments for all potential confounding factors showed that participants with controlled hypertension were not more likely to have RVO than participants without hypertension (aOR = 2.03, 95% CI: 0.94–4.41). However, participants with uncontrolled hypertension, including both stage 1 and stage 2 hypertension (aOR = 3.46, 95% CI: 1.72–6.94), had significantly more RVO than participants without hypertension (stage 1 hypertension (aOR = 2.76, 95% CI: 1.14–5.51) and stage 2 hypertension (aOR = 6.84, 95% CI: 2.36–19.83)).

**Table 4:**
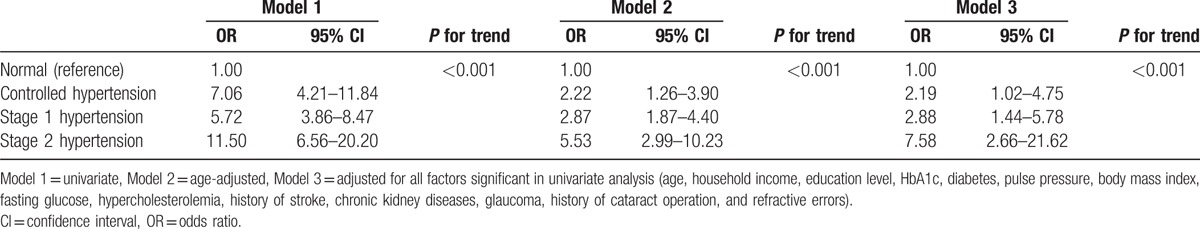
Odds ratios and 95% confidence intervals of retinal vein occlusion in patients with hypertension according to hypertension control.

Table 5 shows the RVO associations according to hypertension control and antihypertensive medication. Multivariate logistic regression analysis with adjustments for all potential confounding factors showed no significant difference in likelihood of RVO between participants treated with antihypertensive medication and normal participants (aOR = 1.51, 95% CI: 0.72–3.17 in patients with hypertension controlled by medication, aOR = 1.02, 95% CI: 0.35–3.00 in patients with hypertension uncontrolled by medication), whereas hypertensive participants who were not taking medication had a significantly higher likelihood of RVO than normal participants (aOR = 3.08, 95% CI: 1.53–6.21).

**Table 5:**
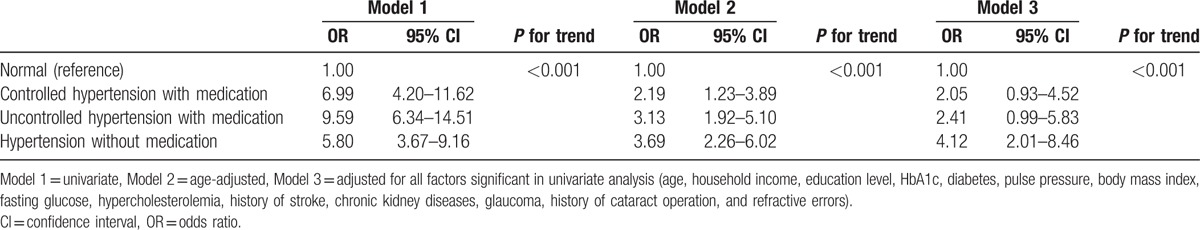
Odds ratios and 95% confidence intervals of retinal vein occlusion in patients with hypertension according to hypertension medication.

## 4 Discussion

The population-based studies of RVO have mostly been performed in white populations. The Blue Mountains Eye Study (BMES) in Australia and the Beaver Dam Eye Study (BDES) reported RVO prevalences of 1.6% and 0.8%, respectively.^[1,5]^ More recently, population-based epidemiologic studies on RVO in nonwhite populations have been published. The RVO prevalence in Asian populations older than 40 years was 0.7% in the Singapore Malay Eye Study (SiMES),^[8]^ 1.2% in the Beijing Eye Study,^[7]^ 2.1% in the Hisayama Study,^[10]^ 0.7% in the Central India Eye and Medical Study (CIEMS),^[11]^ and 0.72% in the Singapore Epidemiology of Eye Disease Study (SEEDS).^[13]^ Our study showed an RVO prevalence of 0.6% (0.6% in BRVO and <0.1% in CRVO) in adults ≥19 years old and 1.0% (0.9% in BRVO and <0.1% in CRVO) in adults ≥40 years old, with no significant sex differences. Our prevalence estimates are moderate compared to the previous results. These variations in RVO prevalence might be due to racial, environmental, or methodological differences. Rogers et al^[9]^ summarized RVO prevalence using pooled data from worldwide studies and concluded that age- and sex-standardized RVO prevalence were highly variable, according to ethnicity (highest in Asians and Hispanics and lowest in whites). Some studies have used epidemiologic data obtained from specified areas, such as rural or urban communities, while our data were obtained from a nationwide health survey, thereby reflecting a representative Korean population. Our study also had the largest sample size among all studies.^[8,10,11]^

In this study, we assessed all RVO risk factors that had been significant in previously published population-based studies. Of demographic and socioeconomic factors, only old age was found to be an independent risk factor of RVO in our study. Old age has consistently been found to be one of the major RVO risk factors across many studies.^[1,5,6,8]^ Our study revealed that RVO prevalence was very low in younger participants (lower than 0.1% ≤40 years) and increased with age. In particular, CRVO was rare in people <70 years old and was observed more frequently in older age groups compared to BRVO. This indicates that RVO is an age-associated disease, and that functional and structural changes in the retinal vessels as a function of aging contribute to RVO pathogenesis. Current smoking was reported to be an RVO-associated factor in the BDES, but we did not find the same pattern in our study.^[8,10]^ No population-based studies, including this one, have identified alcohol drinking as an important RVO risk factor.^[1,5–8,10,11,13]^

Previous studies have reported that RVO shared several risk factors with cardiovascular events (coronary heart diseases, angina, and stroke), such as hypertension, diabetes, smoking, and hypercholesterolemia.^[1,5,6,8,22,23]^ According to our analyses, hypertension presence, hypercholesterolemia, and stroke history were significantly associated with RVO. Hypertension has consistently been identified as an RVO risk factor among many previous studies, except the SiMES.^[8]^ Diabetes was not a significant risk factor according to our study. Histories of angina, myocardial infarction, and stroke and the presence of hypercholesterolemia have been inconsistently reported as RVO risk factors across several studies. Sample size, racial differences, and environmental factors might influence any of the other known risk factors. According to SEEDS, there were some differences in significance for RVO factors among 3 Asian ethnicities studied (Chinese, Indian, and Malay).

Most previous population-based studies did not find an association between stroke history and RVO, except the BMES.^[1,5–8,10–13]^ In contrast to previous studies, our multivariate logistic regression analysis showed that history of stroke was a significant risk factor of RVO. Recently, other studies have also documented a higher stroke risk among RVO patients compared to non-RVO patients.^[24–27]^ On the other hand, while no studies have evaluated the risk of developing RVO after stroke, our data support the conclusion that RVO and stroke are risk factors of each other, perhaps because the retina extends embryonically from the brain, and retinal vessels share anatomical and functional features with cerebral vessels (e.g., the blood–retinal barrier is analogous to the blood–brain barrier).^[28]^

We evaluated the association between other potential biochemical markers (hematocrit, serum ferritin, WBC count, and vitamin D) and RVO. According to the Hisayama study,^[10]^ a higher plasma hematocrit level, which could increase blood viscosity, was reported to be an independent risk factor of RVO. Recently, abnormal serum ferritin level, WBC count, and vitamin D were found to be associated with metabolic syndrome and cardiovascular diseases.^[29–31]^ Our study, however, did not show any significant associations between these factors and RVO.

We did not find a significant independent relationship between metabolic syndrome and RVO. Our study revealed that, among the components of metabolic syndrome, only blood pressure was significantly associated with RVO; this indicates that hypertension is the most important metabolic disorder in the development of RVO. Therefore, we assessed the association between hypertension and RVO in detail. Among hypertensive participants, the OR for RVO increased with hypertension grade. Participants with stage 2 hypertension had an RVO OR more than 7 times greater than that of those without hypertension. Interestingly, our study found that, if participants with hypertension were taking antihypertensive medication, the RVO OR was not significantly higher than that of participants without hypertension, whereas untreated hypertension was significantly associated with RVO (aOR = 4.12, 95% CI: 2.01–8.46). We assumed that if, once patients began taking antihypertensive medication, blood pressure was lower than the initial pressure before medication, there was a period during which blood pressure was dropping even if the patient's blood pressure was measured as being high at the survey time. Although several types of data deficiencies limited our analyses, such as longitudinal blood pressure, duration of hypertension, and types of antihypertensive drugs taken, our results imply that hypertension control and antihypertensive medication use are crucial for preventing RVO. Previously, the BDES compared the association between treated or untreated hypertension and RVO and reported that treated hypertension was significantly associated with RVO (OR = 6.85, 3.79, 10.24 in untreated, treated controlled, and treated uncontrolled groups, respectively) compared to normotension, which was different from our findings.^[1]^ However, these findings come from age-adjusted logistic regression analysis, and the study used a different definition for hypertension than we did (systolic blood pressure ≥160 mm Hg or diastolic pressure ≥95 mm Hg). Additionally, our data might reflect the effectiveness of modern antihypertensive medications because the BDES was performed more than 25 years ago.

Our multivariate regression analysis showed that no ophthalmologic factors were associated with RVO. Glaucoma is known as a risk factor of RVO according to many previous small studies, as well as the BMES.^[5,32,33]^ However, other population-based studies (BDES, SiMES, Beijing Eye Study, and CIEMS) have suggested that glaucoma does not have a significant relationship with RVO prevalence, which was consistent with our study.^[1,7,8,11]^ In CIEMS and SEEDS, various ocular factors were evaluated; however, only a narrow anterior chamber angle was significantly associated with RVO according to the CIEMS^[11,13]^ Both the BDES and the SiMES revealed that higher ocular perfusion pressure was associated with RVO, while the Beijing Eye Study, as well our study, did not find a significant association between ocular perfusion pressure and RVO.^[1,8,34]^ Further studies are required to clarify which ocular factors are significantly associated with RVO.

Several issues and limitations should be considered when interpreting our data. First, we could not determine any causal relationships between risk factors and RVO because ours was a cross-sectional study. A prospective longitudinal cohort study would help to identify causal risk factors. Second, it is possible that we underestimated RVO prevalence because only fundus photographs centered on the macula were evaluated; this could have resulted in missed cases of peripheral RVO. Third, we did not divide RVO into BRVO and CRVO for identifying potential risk factors because sample sizes for CRVO were too small to perform robust statistical analyses. Despite these limitations, a notable strength of our study was its large sample size compared to the other population-based survey studies. Most previous studies enrolled <50 subjects. Furthermore, most other studies only included participants >40 years old, but our study included all adult age groups (≥19 years old), and we presented prevalence rates for the young age groups.^[1,5,7,8,10,11,13]^ Third, the KNHANES was a government-initiated study, thus requiring that all aspects of the survey were performed using the standardized protocol and well-trained examiners, which produced qualified and validated health data from a representative Korean population.

In conclusion, we found a moderate RVO prevalence compared to other studies. Conventional risk factors, such as old age, hypertension, hypercholesterolemia, and history of stroke, were also analyzed in the representative Korean population. Hypertension, a particularly modifiable risk factor, was the most strongly associated factor for RVO in our study. Our results provide supporting evidence that well-controlled hypertension and use of antihypertensive medication protect against RVO occurrence, and that ophthalmologists should pay attention to hypertension control as part of their ophthalmologic treatment plan.

## References

[1] Klein R Klein BE Moss SE The epidemiology of retinal vein occlusion: the Beaver Dam Eye Study. *Trans Am Ophthalmol Soc* 2000; 98:133–141.discussion 141–3. 11190017 PMC1298220

[2] McIntosh RL Rogers SL Lim L Natural history of central retinal vein occlusion: an evidence-based systematic review. *Ophthalmology* 2010; 117:1113.e15–1123.e15. 20430446 10.1016/j.ophtha.2010.01.060

[3] Rogers SL McIntosh RL Lim L Natural history of branch retinal vein occlusion: an evidence-based systematic review. *Ophthalmology* 2010; 117:1094.e5–1101.e5. 20430447 10.1016/j.ophtha.2010.01.058

[4] Rehak J Rehak M Branch retinal vein occlusion: pathogenesis, visual prognosis, and treatment modalities. *Curr Eye Res* 2008; 33:111–131. 18293182 10.1080/02713680701851902 PMC2430176

[5] Mitchell P Smith W Chang A Prevalence and associations of retinal vein occlusion in Australia. The Blue Mountains Eye Study. *Arch Ophthalmol* 1996; 114:1243–1247. 8859084 10.1001/archopht.1996.01100140443012

[6] Wong TY Larsen EK Klein R Cardiovascular risk factors for retinal vein occlusion and arteriolar emboli: the Atherosclerosis Risk in Communities & Cardiovascular Health studies. *Ophthalmology* 2005; 112:540–547. 15808241 10.1016/j.ophtha.2004.10.039

[7] Liu W Xu L Jonas JB Vein occlusion in Chinese subjects. *Ophthalmology* 2007; 114:1795–1796. 17822995 10.1016/j.ophtha.2007.03.010

[8] Lim LL Cheung N Wang JJ Prevalence and risk factors of retinal vein occlusion in an Asian population. *Br J Ophthalmol* 2008; 92:1316–1319. 18684751 10.1136/bjo.2008.140640

[9] Rogers S McIntosh RL Cheung N The prevalence of retinal vein occlusion: pooled data from population studies from the United States, Europe, Asia, and Australia. *Ophthalmology* 2010; 117:313.e1–319.e1. 20022117 10.1016/j.ophtha.2009.07.017 PMC2945292

[10] Yasuda M Kiyohara Y Arakawa S Prevalence and systemic risk factors for retinal vein occlusion in a general Japanese population: the Hisayama study. *Invest Ophthalmol Vis Sci* 2010; 51:3205–3209. 20071683 10.1167/iovs.09-4453

[11] Jonas JB Nangia V Khare A Prevalence and associations of retinal vein occlusions: the Central India Eye and Medical Study. *Retina* 2013; 33:152–159. 22825408 10.1097/IAE.0b013e318260246f

[12] Ponto KA Elbaz H Peto T Prevalence and risk factors of retinal vein occlusion: the Gutenberg Health Study. *J Thromb Haemost* 2015; 13:1254–1263. 25894549 10.1111/jth.12982

[13] Koh V Cheung CY Li X Retinal vein occlusion in a multi-ethnic Asian population: the Singapore Epidemiology of Eye Disease Study. *Ophthalmic Epidemiol* 2016; 23:6–13. 26751637 10.3109/09286586.2015.1082604

[14] Chobanian AV Bakris GL Black HR The Seventh Report of the Joint National Committee on Prevention, Detection, Evaluation, and Treatment of High Blood Pressure: the JNC 7 report. *JAMA* 2003; 289:2560–2572. 12748199 10.1001/jama.289.19.2560

[15] Park SJ Lee JH Woo SJ Age-related macular degeneration: prevalence and risk factors from Korean National Health and Nutrition Examination Survey, 2008 through 2011. *Ophthalmology* 2014; 121:1756–1765. 24813632 10.1016/j.ophtha.2014.03.022

[16] Lee WJ Sobrin L Lee MJ The relationship between diabetic retinopathy and diabetic nephropathy in a population-based study in Korea (KNHANES V-2, 3). *Invest Ophthalmol Vis Sci* 2014; 55:6547–6553. 25205863 10.1167/iovs.14-15001

[17] Jee D Lee WK Kang S Prevalence and risk factors for diabetic retinopathy: the Korea National Health and Nutrition Examination Survey 2008–2011. *Invest Ophthalmol Vis Sci* 2013; 54:6827–6833. 24065813 10.1167/iovs.13-12654

[18] Alberti KG Eckel RH Grundy SM Harmonizing the metabolic syndrome: a joint interim statement of the International Diabetes Federation Task Force on Epidemiology and Prevention; National Heart, Lung, and Blood Institute; American Heart Association; World Heart Federation; International Atherosclerosis Society; and International Association for the Study of Obesity. *Circulation* 2009; 120:1640–1645. 19805654 10.1161/CIRCULATIONAHA.109.192644

[19] National Kidney Foundation. K/DOQI clinical practice guidelines for chronic kidney disease: evaluation, classification, and stratification. *Am J Kidney Dis* 2002; 39 (2 suppl 1):S1–S266. 11904577

[20] Nickolas TL Frisch GD Opotowsky AR Awareness of kidney disease in the US population: findings from the National Health and Nutrition Examination Survey (NHANES) 1999 to 2000. *Am J Kidney Dis* 2004; 44:185–197. 15264176 10.1053/j.ajkd.2004.04.023

[21] Foster PJ Buhrmann R Quigley HA The definition and classification of glaucoma in prevalence surveys. *Br J Ophthalmol* 2002; 86:238–242. 11815354 10.1136/bjo.86.2.238 PMC1771026

[22] Lee JY Yoon YH Kim HK Baseline characteristics and risk factors of retinal vein occlusion: a study by the Korean RVO Study Group. *J Korean Med Sci* 2013; 28:136–144. 23341724 10.3346/jkms.2013.28.1.136 PMC3546092

[23] Di Capua M Coppola A Albisinni R Cardiovascular risk factors and outcome in patients with retinal vein occlusion. *J Thromb Thrombolysis* 2010; 30:16–22. 19705255 10.1007/s11239-009-0388-1

[24] Rim TH Kim DW Han JS Retinal vein occlusion and the risk of stroke development: a 9-year nationwide population-based study. *Ophthalmology* 2015; 122:1187–1194. 25726093 10.1016/j.ophtha.2015.01.020

[25] Park SJ Choi NK Yang BR Risk of stroke in retinal vein occlusion. *Neurology* 2015; 85:1578–1584. 26453647 10.1212/WNL.0000000000002085

[26] Werther W Chu L Holekamp N Myocardial infarction and cerebrovascular accident in patients with retinal vein occlusion. *Arch Ophthalmol* 2011; 129:326–331. 21402990 10.1001/archophthalmol.2011.2

[27] Ho JD Liou SW Lin HC Retinal vein occlusion and the risk of stroke development: a five-year follow-up study. *Am J Ophthalmol* 2009; 147:283.e2–290.e2. 18835470 10.1016/j.ajo.2008.08.006

[28] Tso MO Jampol LM Pathophysiology of hypertensive retinopathy. *Ophthalmology* 1982; 89:1132–1145. 7155524 10.1016/s0161-6420(82)34663-1

[29] Lee YJ Shin YH Kim JK Metabolic syndrome and its association with white blood cell count in children and adolescents in Korea: the 2005 Korean National Health and Nutrition Examination Survey. *Nutr Metab Cardiovasc Dis* 2010; 20:165–172. 19616924 10.1016/j.numecd.2009.03.017

[30] Jehn M Clark JM Guallar E Serum ferritin and risk of the metabolic syndrome in U.S. adults. *Diabetes Care* 2004; 27:2422–2428. 15451911 10.2337/diacare.27.10.2422

[31] Kang HT Linton JA Shim JY Serum ferritin level is associated with the prevalence of metabolic syndrome in Korean adults: the 2007–2008 Korean National Health and Nutrition Examination Survey. *Clin Chim Acta* 2012; 413:636–641. 22212623 10.1016/j.cca.2011.12.011

[32] Kim MJ Woo SJ Park KH Retinal nerve fiber layer thickness is decreased in the fellow eyes of patients with unilateral retinal vein occlusion. *Ophthalmology* 2011; 118:706–710. 21055813 10.1016/j.ophtha.2010.08.028

[33] Risk factors for central retinal vein occlusion. The Eye Disease Case-Control Study Group. *Arch Ophthalmol* 1996; 114:545–554. 8619763

[34] Zhou JQ Xu L Wang S The 10-year incidence and risk factors of retinal vein occlusion: the Beijing eye study. *Ophthalmology* 2013; 120:803–808. 23352194 10.1016/j.ophtha.2012.09.033

